# Comprehensive anticancer drug response prediction based on a simple cell line-drug complex network model

**DOI:** 10.1186/s12859-019-2608-9

**Published:** 2019-01-22

**Authors:** Dong Wei, Chuanying Liu, Xiaoqi Zheng, Yushuang Li

**Affiliations:** 1 School of Science, Yanshan University, Qinhuangdao, 066004 China; 2 Department of Mathematics, Shanghai Normal University, Shanghai, 200234 China

**Keywords:** Anticancer drug response, Cell line-drug complex network, Computational prediction model, Cell line, Precision medicine

## Abstract

**Background:**

Accurate prediction of anticancer drug responses in cell lines is a crucial step to accomplish the precision medicine in oncology. Although many popular computational models have been proposed towards this non-trivial issue, there is still room for improving the prediction performance by combining multiple types of genome-wide molecular data.

**Results:**

We first demonstrated an observation on the CCLE and GDSC datasets, i.e., genetically similar cell lines always exhibit higher response correlations to structurally related drugs. Based on this observation we built a cell line-drug complex network model, named CDCN model. It captures different contributions of all available cell line-drug responses through cell line similarities and drug similarities. We executed anticancer drug response prediction on CCLE and GDSC independently. The result is significantly superior to that of some existing studies. More importantly, our model could predict the response of new drug to new cell line with considerable performance. We also divided all possible cell lines into “sensitive” and “resistant” groups by their response values to a given drug, the prediction accuracy, sensitivity, specificity and goodness of fit are also very promising.

**Conclusion:**

CDCN model is a comprehensive tool to predict anticancer drug responses. Compared with existing methods, it is able to provide more satisfactory prediction results with less computational consumption.

**Electronic supplementary material:**

The online version of this article (10.1186/s12859-019-2608-9) contains supplementary material, which is available to authorized users.

## Background

The inherent heterogeneity of cancers always makes the same cancer patients exhibiting different anticancer drug responses, which is a major difficulty in cancer treatment. It is critical to accurately predict the therapy responses of patients based on their molecular and clinical profiles [1, 2]. With the rapid development of high-throughput technology, a huge number of publicly available cancer genomic data have been generated by large research agencies. It supplies a golden opportunity to translate massive data into knowledge of tumor biology and then improve anticancer drug response prediction. Many computational methods have greatly contributed to this non-trivial issue [3–6]. Supervised learning technique is one of the most widely used approaches. It can be mainly partitioned into regression and classification models [7]. The former always generate numerical estimations of drug sensitivity represented by activity area or IC50 [3, 8], and the latter tend to make a high or low sensitivity prediction depending on the predetermined response levels [9, 10]. Machine learning tools to implement these methods include support vector machines [11], random forests [12], neural network [4] and logistic ridge regression [13]. Comparative analysis suggested that regression model, such as elastic net and ridge regression, exhibit good and robust performance in different settings [9, 14].

Besides the above two types of methods, another important method that gains much attention is the network-based models [15–19]. One of the earliest attempts should be traced back to Zhang et al. [20], who presented a dual-layer integrated cell line-drug network model by combining the predictions from the individual layers. Reader could refer to [7, 9, 21] for grasping more computational approaches.

Although achieving promising results for certain drugs, most models focused on predicting three types of responses, i.e., ‘old drug to old cell line’, ‘old drug to new cell line’ and ‘new drug to old cell line’ (here ‘old’ means tested or existed, and ‘new’ means untested), but paid less attention to the response prediction of ‘new drug to new cell line’. As we all know, updating an existing cancer screen with the latest available drugs and cell lines is not a trivial issue, because it always requires the same expertise, infrastructure and conditions as when the screen was accomplished the first time around. In addition, comprehensive prediction might make potential cancer screen more accurate and experimental design more flexible, as well as accelerate early drug evaluation. Such efforts should be greatly aided by accurate preclinical computational methods.

To predict the response of ‘new drug to new cell line’, we should take advantage of all observed (tested or existed) cell line-drug response values. Importantly, two questions need to be asked. The first is whether observed response values have statistical power to predict the response of ‘new drug to new cell line’. The second is how to evaluate the prediction performance of the proposed model. We aim to answer the above two questions.

Shivakumar et al. found that structural similarity between drug pairs in the NCI-60 dataset highly correlates with the similarity between their activities across the cancer cell lines [22]. Zhang et al. showed that genetically similar cell lines may also respond very similarly to a given drug, and structurally related drugs may have similar responses to a given cell line [20]. We are wondering whether their ideas could be extended to a more general circumstance, that is, genetically similar cell lines always exhibit higher response correlations to structurally related drugs. If it is true, we aim to construct a cell line-drug complex network (CDCN) model which incorporates cell line similarity and drug similarity information, as well as cell line-drug responses. To answer the second question, we executed CDCN model on the Cancer Cell Line Encyclopedia (CCLE) [23] and the Genomics of Drug Sensitivity in Cancer (GDSC) [24] datasets respectively, and obtained the satisfactory prediction result. Besides inputting missing values of drug response data, we also classified cell lines into sensitive group and resistant group according to the observed response to a given drug. The prediction accuracy, sensitivity, specificity and goodness of fit further justified the good performance of our model.

## Methods

### Data and preprocessing

**Fig. 1:**
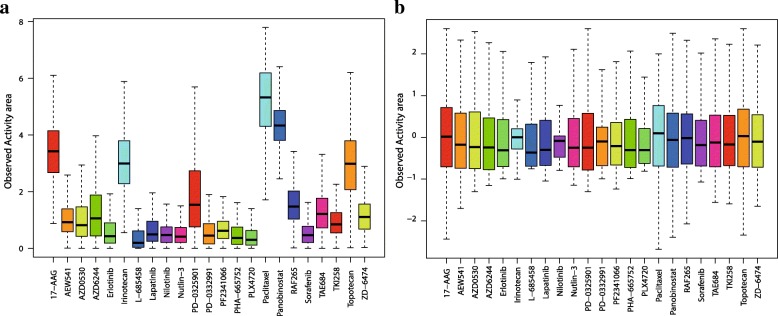
Normalization of drug response data for CCLE dataset. (**a**) The primary data. (**b**) Normalized data

Cancer Cell Line Encyclopedia (CCLE) [23] and Genomics of Drug Sensitivity in Cancer (GDSC) project [24] are two most important resources of publicly available data for investigating anticancer drug response. They are benchmark compilations of gene expression, gene copy number and massively parallel sequencing data. We selected 491 cancer cell lines from CCLE, downloaded the chemical structure files of 23 drugs from PubChem Compound, and then obtained a cell line-drug response matrix consisting of 11,293 entries, of which 423 (3.75%) are missing values. We also selected 655 cancer cell lines from GDSC and 129 drugs in the PubChem database. The resulting drug response matrix has 84,495 entries, out of which 15,763 (18.66%) are missing. The given drug responses were measured by activity area for CCLE and IC50 for GDSC. Higher Activity area or lower IC50 value indicates a better sensitivity of the cell line to a given drug. To eliminate the differences in susceptibility of different drugs, we normalized the drug response data such that all cell line susceptibility data have the same baseline and the same range (see Fig. 1 as an example).

### Generalized observation

For the first question, we want to know whether available drug-cell line response values have the statistical power to predict the response of ‘new drug to new cell line’. Motivated by [20, 22], we first examined the response correlations between genetically similar cell lines and structurally similar drugs.

Cell line similarities are measured by Pearson correlation coefficients between their corresponding gene expression profiles. The correlations of most cell line pairs (around 92% for CCLE, 70% for GDSC) are larger than 0.8. We divided all possible cell line pairs with correlation coefficients higher than 0.9 into high similar group ‘Hc’, and other pairs into low similar group ‘Lc’.

**Fig. 2:**
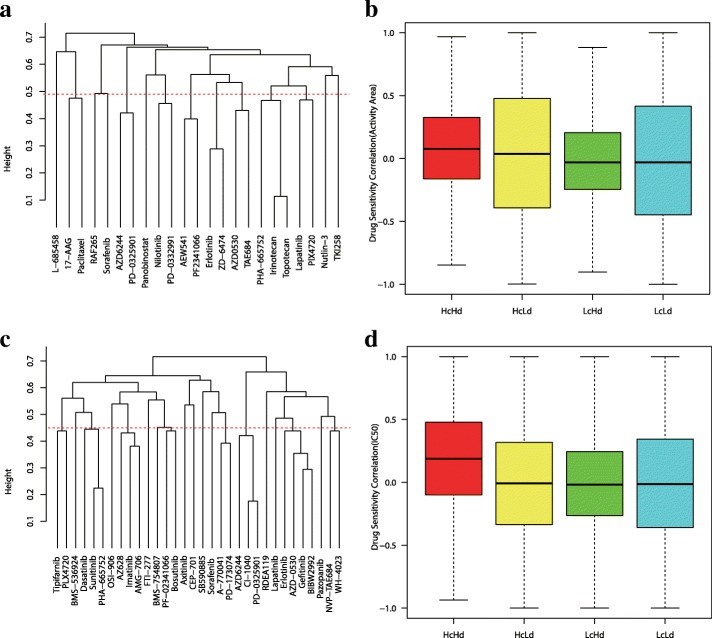
Model assumption. (**a**) A cluster of 23 drugs in CCLE. (**c**) A cluster of 32 drugs in GDSC. (**b**) and (**d**) show a general observation: similar cell lines have higher response correlations to similar drugs. The X-axis shows four combinations of two cell line groups and two drug groups. The Y-axis shows the correlations of drug responses between cell line pairs

Next, we used Open Babel to obtain molecular fingerprints of selected drugs [25]. Fingerprint-based Tanimoto coefficient is often used as a molecular similarity indicator in cheminformatics literature [22, 26, 27]. Define the distance between two drugs as *d*(D_*i*_, D_*j*_) = 1 − T(D_*i*_, D_*j*_), where T(D_*i*_, D_*j*_) is the Tanimoto coefficient between drugs D_*i*_ and D_*j*_. Based on the drug distance matrix (see Additional file 1: Table S1 and Additional file 2: Table S2), we clustered all drugs using “complete” method in R. Drugs with high distances tend to be in different clusters, while drugs with similar structure are expected to be clustered together (see Fig. 2a and c). For CCLE dataset, we extracted such drug pairs from Fig. 2a with Tanimoto coefficient greater than 0.5 and distance less than 0.49 into high similar group ‘Hd’: {17-AAG, Paclitaxel, AZD6244, PD-0325901, Nilotinib, PD-0332991, AEW541, PF2341066, Erlotinib, ZD-6474, AZD0530, TAE684, Lapatinib, PLX4720, PHA-665752, Irinotecan, Topotecan}. Other drug pairs were divided into low similar group ‘Ld’. For GDSC dataset, we extracted such drug pairs from Fig. 2c with Tanimoto coefficient greater than 0.5 and distance less than 0.45 into high similar group ‘Hd’: {Tipifarnib, PLX4720, Dasatinib, Sunitinib, PHA-665752, AZ628, Imatinib, AMG-706, BMS-754807, PF-02341066, Bosutinib, A-770041, PD-173074, AZD6244, CI-1040, PD-0325901, Erlotinib, AZD-0530, Gefitinib, BIBW2992, NVP-TAE684, WH-4023}. Other drug pairs were divided into low similar group ‘Ld’. From Fig. 2b and d we found that more similar Cell lines always show higher response correlations to more similar drugs, it holds for both CCLE and GDSC data sets.

### Construction of cell line-drug complex network model

**Fig. 3:**
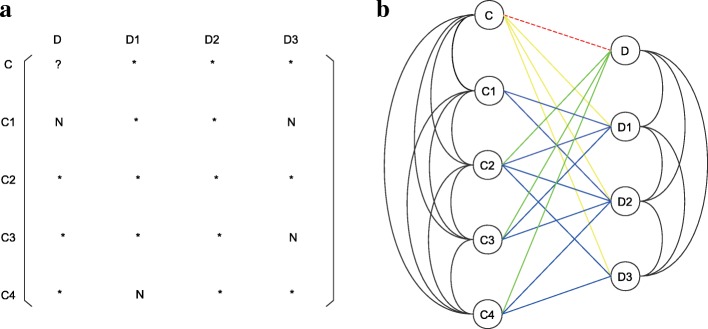
Example of CDCN. (**a**) A cell line-drug response matrix. (**b**) The corresponding cell line-drug complex network. The dotted red line denotes the edge of the pair **c** and **d** on which we focused. Different color lines represent edges of different types of cell line-drug pairs

We use Ω to represent the set of all possible cell line-drug pairs. Denote *ρ*(C, C_*i*_) as the Pearson correlation coefficient between cell lines C and C_*i*_, *T*(D, D_*j*_) as the Tanimoto coefficient between drugs D and D_*j*_. Meanwhile, we use *R*(C, D) to represent the observed response value of the pair (C, D) ∈ Ω. Define C_*i*_ and C_*j*_ as adjacent if *ρ*(C_*i*_, C_*j*_) ≠ 0, and the weight of this edge as *ρ*(C_*i*_, C_*j*_). Similarly, D_*i*_ and D_*j*_ are called adjacent if their weight *T*(D_*i*_, D_*j*_) > 0. Define C_*i*_ and D_*j*_ as adjacent if *R*(C_*i*_, D_*j*_) is available. Obviously, the resulting network involves cell line similarity and drug similarity information, as well as cell line-drug response situations, so we call it the cell line-drug complex network (CDCN). In fact, this network is the dual-layer integrated cell line-drug network in [20]. Figure 3b showed a CDCN corresponding to the cell line-drug response matrix described in Fig. 3a.

Define $w\left(\mathrm{C},{\mathrm{C}}_i\right)={e}^{-\frac{{\left(1-\rho \left(\mathrm{C},{\mathrm{C}}_i\right)\right)}^2}{2{\alpha}^2}}$ as a weight function of cell lines. It increases with respect to *ρ*(C, C_*i*_), where the parameter *α* measures the decay rate with the decrease of *ρ*(C, C_*i*_). Similarly, define a weight function of drugs $w\left(\mathrm{D},{\mathrm{D}}_j\right)={e}^{-\frac{{\left(1-T\left(\mathrm{D},{\mathrm{D}}_j\right)\right)}^2}{2{\tau}^2}}$ with decay parameter *τ*.

For a given pair (C, D), let Ω\{(C, D)} be the set of all other pairs (C_*i*_, D_*j*_) besides (C, D). Based on the generalized observation we are able to make a prediction by dealing with all possible observed response values *R*(C_*i*_, D_*j*_) as the following,

1 $$ \hat{R}\left(\mathrm{C},\mathrm{D}\right)=\frac{\sum_{\left({\mathrm{C}}_i,{\mathrm{D}}_j\right)\in \Omega \setminus \left\{\left(\mathrm{C},\mathrm{D}\right)\right\}}w\left(\mathrm{C},{\mathrm{C}}_i\right)w\left(\mathrm{D},{\mathrm{D}}_j\right)R\left({\mathrm{C}}_i,{\mathrm{D}}_j\right)}{\sum_{\left({\mathrm{C}}_i,{\mathrm{D}}_j\right)\in \Omega \setminus \left\{\left(\mathrm{C},\mathrm{D}\right)\right\}}w\left(\mathrm{C},{\mathrm{C}}_i\right)w\left(\mathrm{D},{\mathrm{D}}_j\right)} $$

where $\widehat{R}\left(\mathrm{C},\mathrm{D}\right)$ is the predicted response value for the pair (C, D). The product *w*(C, C_*i*_)*w*(D, D_*j*_) reflects the contribution of *R*(C_*i*_, D_*j*_) to $\widehat{R}\left(\mathrm{C},\mathrm{D}\right)$.

**Fig. 4:**
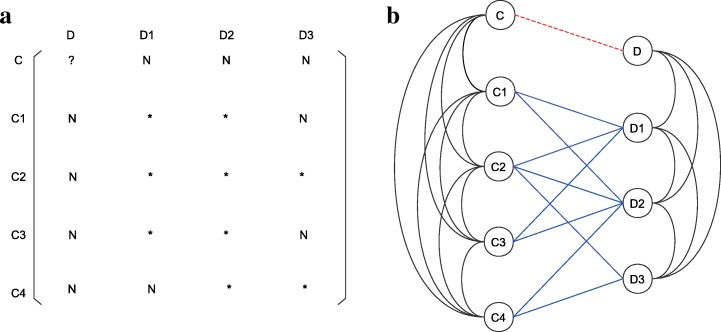
Example of reduced CDCN. (**a**) A reduced cell line-drug response matrix. (**b**) The corresponding reduced cell line-drug complex network

It is worth mentioning that formula (1) is applicable to all types of pairs (C, D). Even if C and D are both new (it means that *R*(C, D_*j*_) and *R*(C_*i*_, D) are not known for any existing drug D_*j*_ and any existing cell line C_*i*_). In this circumstance, the cell line-drug response matrix and the corresponding cell line-drug complex network showed in Fig. 3 would be changed into ones depicted in Fig. 4. Formula (1) also has a ‘little variation’ in the assignment of the pair (C_*i*_, D_*j*_), that is

2 $$ \hat{R}\left(\mathrm{C},\mathrm{D}\right)=\frac{\sum_{\begin{array}{c}\left({\mathrm{C}}_i,{\mathrm{D}}_j\right)\in \Omega \\ {}{\mathrm{C}}_i\ne \mathrm{Cand}{\mathrm{D}}_j\ne \mathrm{D}\end{array}}w\left(\mathrm{C},{\mathrm{C}}_i\right)w\left(\mathrm{D},{\mathrm{D}}_j\right)R\left({\mathrm{C}}_i,{\mathrm{D}}_j\right)}{\sum_{\begin{array}{c}\left({\mathrm{C}}_i,{\mathrm{D}}_j\right)\in \Omega \\ {}{\mathrm{C}}_i\ne \mathrm{Cand}{\mathrm{D}}_j\ne \mathrm{D}\end{array}}w\left(\mathrm{C},{\mathrm{C}}_i\right)w\left(\mathrm{D},{\mathrm{D}}_j\right)} $$

The ‘little variation’ is crucial for accomplishing the response prediction of ‘new drug to new cell line’. To highlight the difference between two formulas, we called formula (1) as CDCN model I and formula (2) as CDCN model II.

The decay parameter pairs (*α*, *τ*) could be optimized by minimizing the following overall error function

3 $$ \left(\widehat{\alpha},\widehat{\tau}\right)={\mathrm{argmin}}_{\left(\alpha, \tau \right)}{\sum}_{\left(\mathrm{C},\mathrm{D}\right)\in \Omega}{\left(\widehat{R}\left(\mathrm{C},\mathrm{D}\right)-R\left(\mathrm{C},\mathrm{D}\right)\right)}^2 $$

where *α* and *τ* are ranged from 0 to 1 with increment 0.01, respectively, and the pair (*α*, *τ*) takes all possible combinations.

We conducted leave-one-out cross-validation by singling out each cell line-drug pair as the test dataset, and used Pearson correlation coefficients between predicted and observed response values to evaluate the predictive power of the proposed model. Root mean square error (RMSE) and normalized root mean square error (NRMSE) of each drug D were also calculated to assess the model.

4 $$ \mathrm{RMSE}\left(\mathrm{D}\right)=\sqrt{\frac{\sum_{\mathrm{C}}{\left(\widehat{R}\left(\mathrm{C},\mathrm{D}\right)-R\left(\mathrm{C},\mathrm{D}\right)\right)}^2}{n}} $$

5 $$ \mathrm{NRMSE}\left(\mathrm{D}\right)=\frac{\mathrm{RMSE}\left(\mathrm{D}\right)}{\max_{\mathrm{C}}R\left(\mathrm{C},\mathrm{D}\right)-{\min}_{\mathrm{C}}R\left(\mathrm{C},\mathrm{D}\right)} $$

Where C ranges over all cell lines for which *R*(C, D) are known, and *n* is the number of such cell lines.

## Results

We executed the following four experiments. (1) Using CDCN model I to predict general responses for the CCLE and GDSC datasets and comparing with six popular computational models. (2) Taking each existed drug-cell line pair as a ‘new drug-new cell line’ pair, we used CDCN model II to predict special responses of these ‘new pairs’, and then compared with the general prediction of model I. (3) Using two models to impute missing data in GDSC independently. (4) Evaluating the model accuracy, sensitivity, specificity and goodness of fit by classifying cell lines into sensitive and resistant groups to some given drug.

### General response prediction

**Fig. 5:**
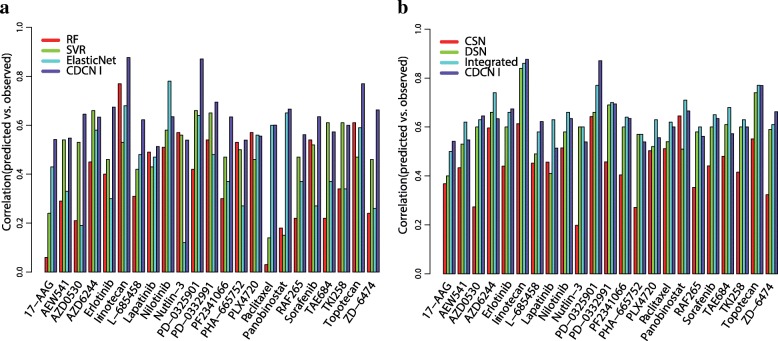
Performance comparisons of seven methods for 23 drugs in CCLE based on Pearson correlations between the predicted and observed activity areas. (**a**) Bar graph showing the prediction performances of RF, SVR, Elastic Net and CDCN I. (**b**) Bar graph showing the prediction performances of CSN, DSN, Integrated and CDCN I

We first applied CDCN model I to the CCLE dataset with the optimized parameters $\left(\widehat{\alpha},\widehat{\tau}\right)=\left(0.02,0.18\right)$. The mean of Pearson correlation coefficients between predicted and observed response values is 0.63 (the minimum is 0.51, the maximum is 0.88). From Fig. 5a, it is evident that our prediction is significantly better than the results by random forest (RF), support vector regression (SVR) and Elastic Net models. Figure 5b showed that CDCN model I is much better than the CSN model (using the cell line similarity network) for all 23 drugs (100%), and DSN model (using the drug similarity network) for 17 drugs (73.91%), also higher than Integrated model (integrating CSN and DSN) for 10 drugs (43.48%). It is anticipated because both CSN and DSN models use less information compared with our model. Meanwhile, Integrated model is an optimal weighted combination of CSN and DSN, which enhanced the prediction performance but greatly restricted its application. In fact, CSN model works for old drugs, and DSN model works for old cell lines. Therefore, Integrated model only works for prediction of old drugs to old cell lines.

**Fig. 6:**
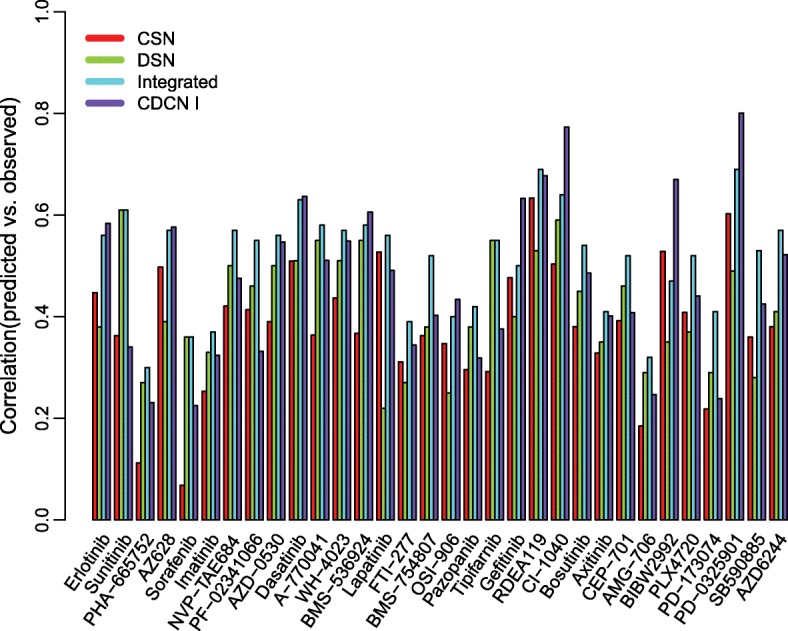
Comparisons of four methods for 32 drugs in GDSC

Next, we conducted CDCN model I for the GDSC dataset with the optimized parameters $\left(\widehat{\alpha},\widehat{\tau}\right)=\left(\mathrm{0.03,0.18}\right)$. Here we focused on 32 drugs targeting genes in the ERK pathways, and compared with CSN, DSN and Integrated models. As can be seen from Fig. 6, Pearson correlations between observed and predicted response values of our model is higher than 0.5 for nearly half of 32 drugs. It is much better than CSN model for 29 drugs (87.88%), DSN for 21 drugs (65.63%), and also than Integrated model for 9 drugs (28.13%).

### Special response prediction

**Fig. 7:**
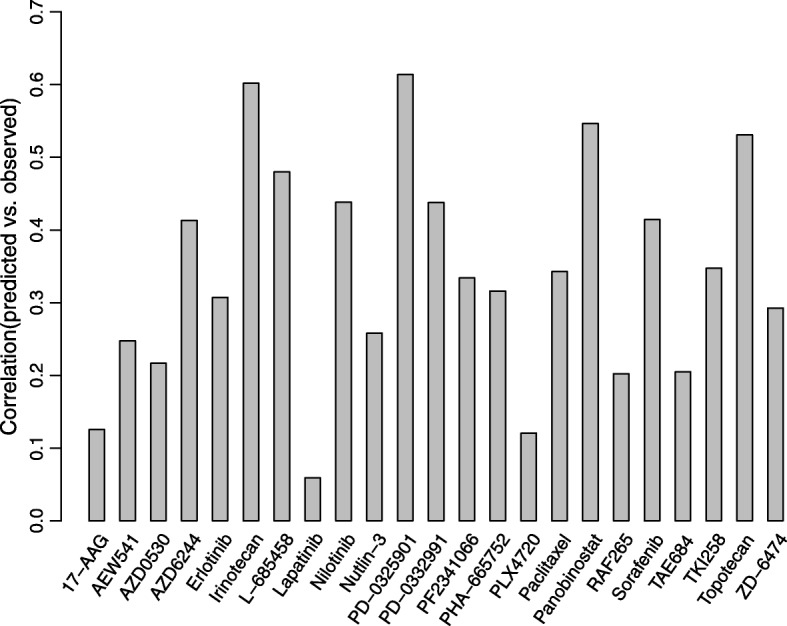
Pearson correlation coefficients between predicted and observed response values for 23 drugs in CCLE using CDCN model II

We used CDCN model II to make a special prediction, i.e. the response prediction of ‘new cell line-new drug’. Fig. 7 summarized Pearson correlation coefficients between predicted and observed response values for the drugs in CCLE with the optimized parameters $\left(\widehat{\alpha},\widehat{\tau}\right)=\left(0.03,0.16\right)$. The correlation coefficients of 9 drugs (39.13%) are higher than 0.4. Specificly, four drugs (Irinotecan, PD-0325901, Panobinostat and Topotecan) exhibit good correlations greater than 0.5.

**Fig. 8:**
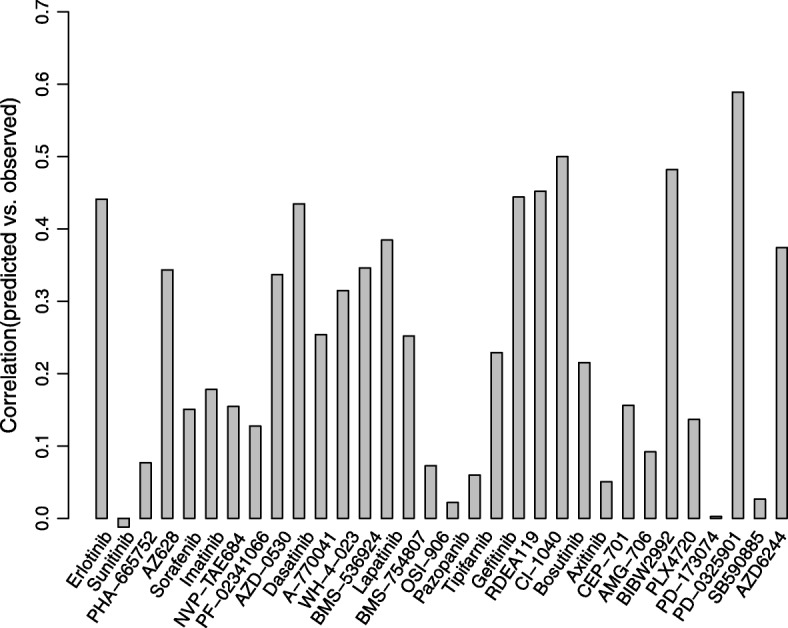
Pearson correlation coefficients between predicted using CDCN model II and observed response values for 32 drugs in GDSC

We also performed special response prediction for 32 drugs in GDSC with the optimized parameters $\left(\widehat{\alpha},\widehat{\tau}\right)=\left(0.04,0.18\right)$, As can be seen from Fig. 8, correlations of seven drugs (21.88%) are greater than 0.4. Four drugs, PD-0325901, RDEA119, CI-1040 and BIBW2992, show higher correlations than 0.45.

**Fig. 9:**
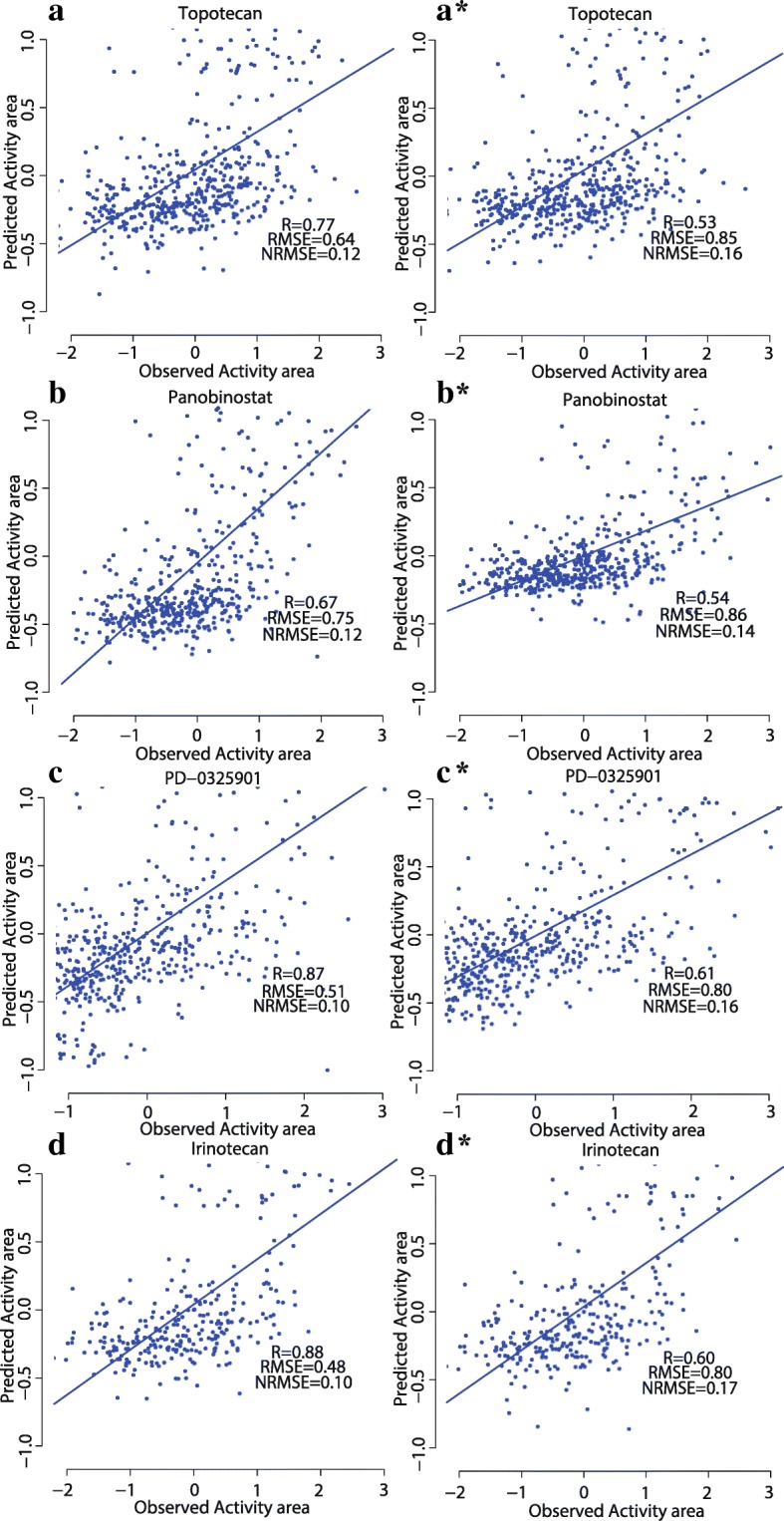
Performance comparisons of CDCN models I and II for 4 drugs in CCLE. (**a**, **b**, **c**, **d**) showing scatter plots of observed and predicted drug responses based on CDCN model I. (A*, B*, C*, D*) showing scatter plots of observed and predicted drug responses based on CDCN model II

**Fig. 10:**
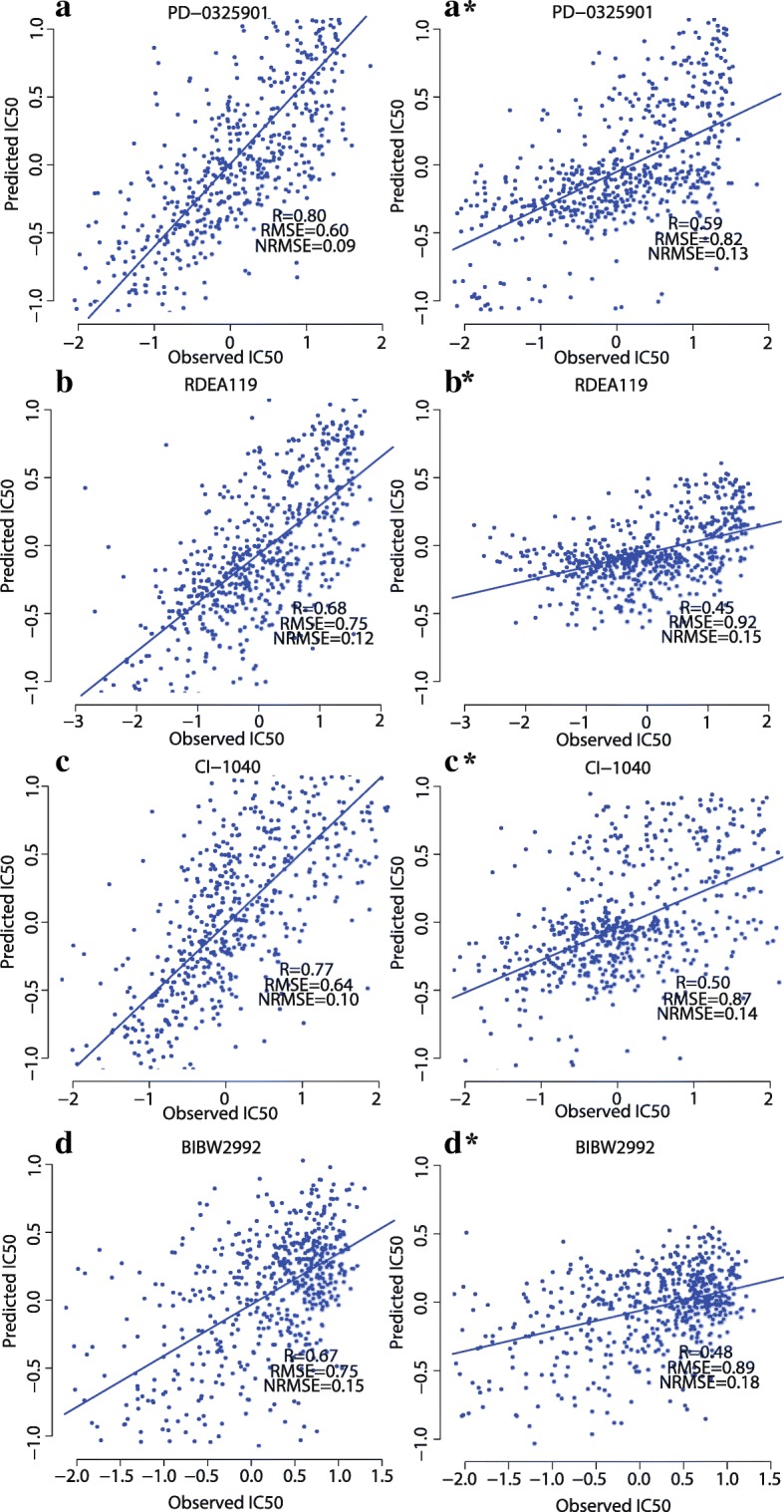
Performance comparisons of CDCN models I and II for 4 drugs in GDSC. (**a**, **b**, **c**, **d**) showing scatter plots of observed and predicted drug responses based on CDCN model I. (A*, B*, C*, D*) showing scatter plots of observed and predicted drug responses based on CDCN model II

Scatter plots in Figs. 9 and 10 suggested that the good correlations are not caused from a small number of outliers. Here, outliers might arise from different aspects. For example, we only used gene expression profile and chemical structures of drugs to build model. Although they are the most widely used sources and powerful features for the drug response investigations, our model still neglected several important information including mutation and copy number variation. Meanwhile, as reported by many researches drug response values are highly inconsistent for some drugs between CCLE and GDSC [11, 28, 29]. These technical noises might be a possible reason for the outliers.

**Fig. 11:**
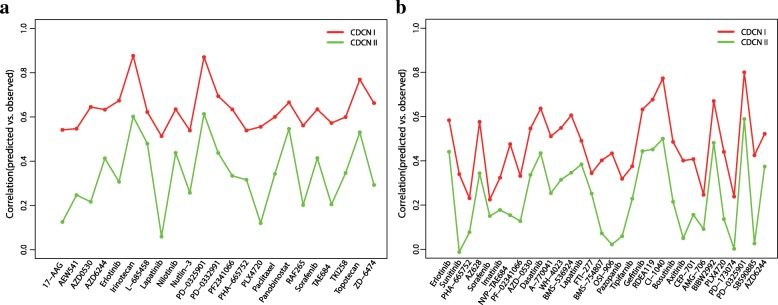
Performance comparison of CDCN models I and II for two datasets. (**a**) Two correlation (between predicted and observed response values) lines based on the CCLE datasets. (**b**) Two correlation (between predicted and observed response values) lines based on the GDSC dataset. The red broken line is the correlation line based on CDCN model I, and the green broken line is the correlation line based on CDCN model II

Obviously, the model II is inferior to model I due to the loss of crucial values such as *R*(C_*i*_, D) and *R*(C, D_*j*_) (see Fig. 11). However, their prediction tendencies are completely consistent except for a few drugs, so model II is a reliable tool for predicting response of ‘new drug-new cell line’.

### Inputting missing data in drug response matrix

**Fig. 12:**
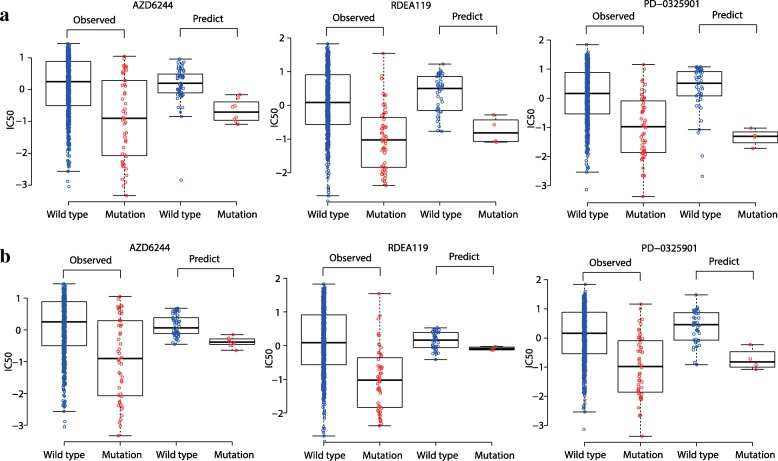
Comparisons between predicted and observed IC50 values for BRAF mutant and wild-type cell lines to three MEK1/2-inhibitors. (**a**) Consistence between the predicted response values by CDCN model I and the observed response values. (**b**) Consistence between the predicted response values by CDCN model II and the observed response values

**Fig. 13:**
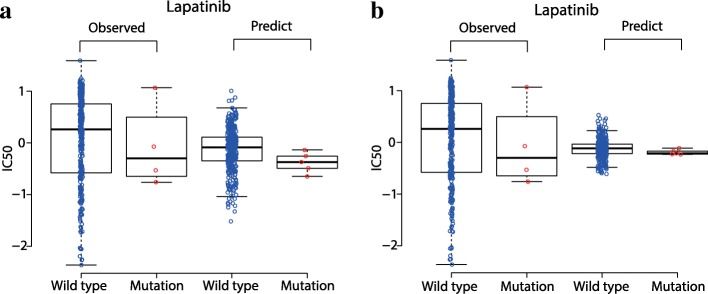
Comparisons between predicted and observed IC50 values for EGFR mutant and wild-type cell lines to Lapatinib. (**a**) Consistence between the predicted response values by CDCN model I and the observed response values. (**b**) Consistence between the predicted response values by CDCN model II and the observed response values

The estimation of missing data is considered to be reliable if they exhibit the same or consistent distribution pattern as that by existing data. Following this definition, we first focused on three MEK inhibitors AZD6244, RDEA119, and PD-0325901 in GDSC dataset. Nearly 7% of response values of these three drugs are missing. We found that the predicted missing response values using CDCN models both have a consistent pattern with the existed (observed) response values. We used fold-change and *P*-value by t.test to illustrate the “consistent pattern” statistically. As is shown in Fig. 12, the observed response values of wild type cell lines are significantly higher than that of BRAF mutated cell lines to three MEK inhibitors AZD6244 (fold-change = 1.26 and *P* = 3.75e-6), RDEA119 (fold-change = 2.02 and *P* = 3.02e-11) and PD-0325901 (fold-change = 1.40 and *P* = 1.61e-9). Consistently, the predicted response values of wild type cell lines are also higher than that of BRAF mutated cell lines to AZD6244 (fold-change = 1.09 and *P* = 6.64e-5 for CDCN model I; fold-change = 0.98 and *P* = 6.07e-7 for CDCN model II), RDEA119 (fold-change = 1.10 and *P* = 4.79e-3 for CDCN model I; fold-change = 1.29 and *P* = 2.91e-5 for CDCN model II) and PD-0325901 (fold-change = 1.35 and *P* = 9.41e-6 for CDCN model I; fold-change = 1.17 and *P* = 3.90e-3 for CDCN model II). In summary, BRAF-mutated cell lines are more sensitive to MEK inhibitors, which is in accordance with the previously published work [20]. Similarly, we also looked at the response difference of the dual kinase inhibitor Lapatinib between EGFR mutated and wild type cell lines. More than half of response values are missing. We found that EGFR-mutated cell lines are more sensitive to Lapatinib (see Fig. 13) which is in agreement with the study [6]. All above results proved that our model could correctly predict drug responses of missing data in GDSC dataset.

**Fig. 14:**
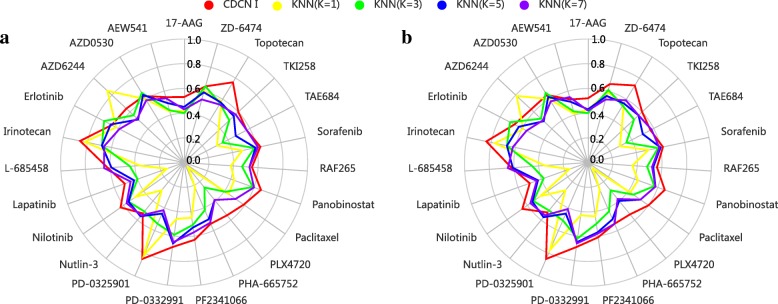
The comparison of predictive performances between CDCN I and kNN methods. (**a**) showing the result on the CCLE dataset with 10% random missing data, and (**b**) showing the result on the CCLE dataset with 20% random missing data

We further compared our method with the nearest neighbor (NN) algorithms in filling up the missing data [30] to justify the performance of our method. In detail, we randomly deleted 10% of response values in CCLE dataset, and performed the CDCN I and kNN models with k = 1, 3, 5 and 7 respectively on the remaining data. Here, the distance between two drugs D_*i*_ and D_*j*_ is defined as 1- *T*(D_*i*_, D_*j*_). We repeated above procedure five times, and used the mean of five Pearson correlation coefficients between predicted and observed response values as the model accuracy. As is shown in Fig. 14a, our model significantly outperforms kNN methods at different values of k. To further verify the robustness of our model, we also randomly deleted 20% of response values in CCLE dataset and obtained similar result as the 10% case (see Fig. 14b).

### Prediction accuracy, sensitivity, specificity and goodness of fit

**Fig. 15:**
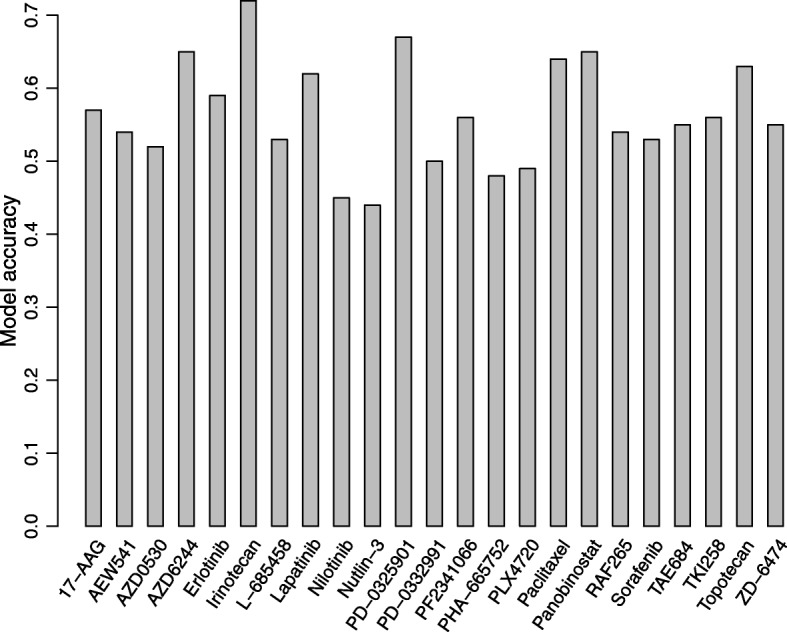
The predicted accuracy for the CCLE dataset

We used a similar method as [11, 31] to evaluate the performance of our model. In detail, for each drug in CCLE, selected the top 200 cell lines with the largest response activity areas to this drug and defined them as the “sensitive” group (if not available, we selected all cell lines with activity area greater than zero as the sensitive group). In contrast, we selected 200 cell lines with the lowest drug responses and defined them as “resistant” group (if not available, we selected all cell lines with activity area less than zero as resistant group). The rest cell lines were considered to be intermediate and eliminated from our analysis. For the prediction results obtained from CDCN I model, we took the same measure as above to assess sensitive and resistant cell lines to the given drug. Figure 15 shows that our model achieved the accuracy of over 60% for 7 of 23 drugs, and over 50% for 19 drugs of 23 drugs. Sensitivity and specificity are over 0.5 for 20 of 23 drugs. Goodness of fit is over 0.2 for 13 of 23 drugs. Additional file 3: Table S3 lists the detail information.

**Fig. 16:**
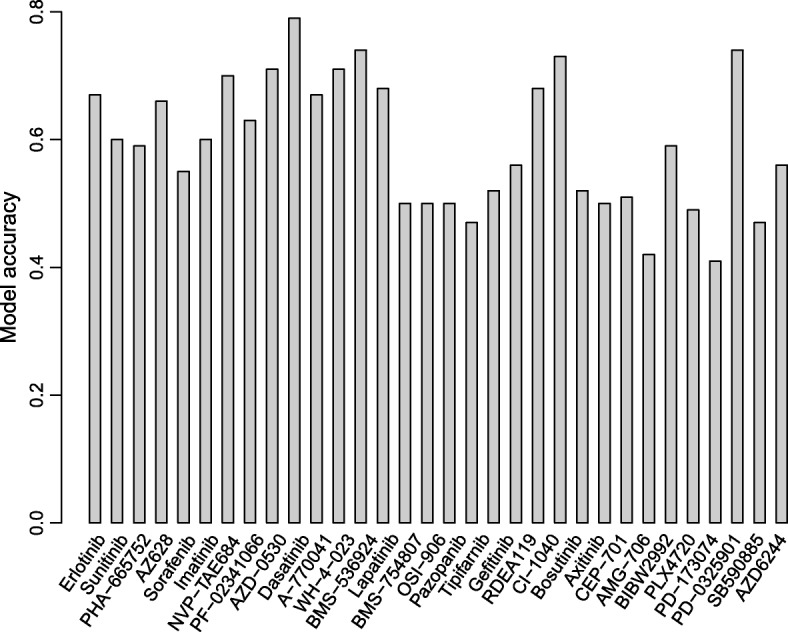
The predicted accuracy for the GDSC dataset

For GDSC dataset, our model accuracy is over 60% for 15 of 32 drugs, and over 50% for 27 drugs (see Fig. 16). Sensitivity and specificity are over 0.5 for 29 of 32 drugs. Goodness of fit is over 0.2 for 16 of 32 drugs, especially for the drug CI.1040 whose goodness of fit is 0.6 and the drug PD-0325901 is 0.64. Additional file 4: Table S4 lists the detail information.

Here we should point out that the goodness of fit is relatively small (lower than 0.2) for around half of drugs in both CCLE and GDSC. It is possible even if our model is satisfactory, because CCLE and GDSC are both cross-section datasets, the goodness of fit may be lower because of the variation between the observed values.

**Fig. 17:**
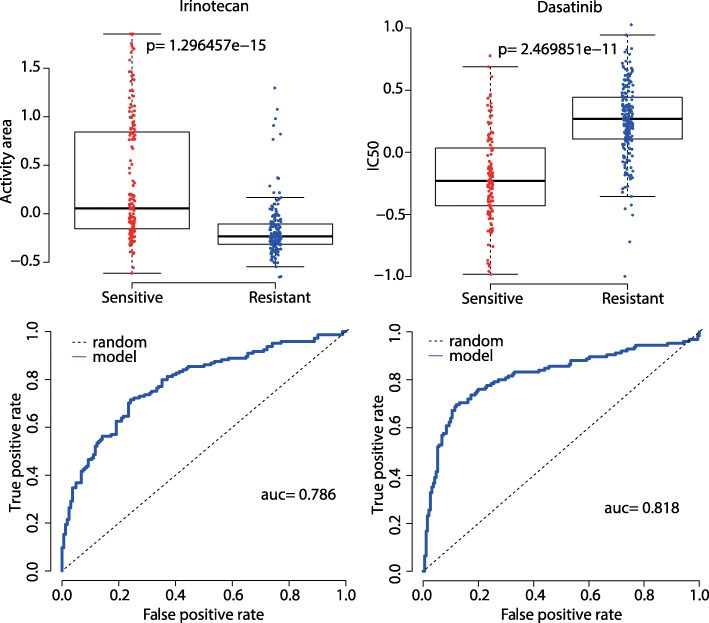
Boxplot and ROC curve (the bottom curve indicates drug response) for Irinotecan in CCLE and Dasatinib in GDSC. The left for Irinotecan, *p*-value by T test is 1.296e-15 and AUC is 0.786. The right for Dasatinib, *p*-value by T test is 2.470e-11 and AUC is 0.818

We further tested our model for Irinotecan in CCLE dataset and Dasatinib in GDSC dataset. As is shown in Fig. 17, our model achieved the AUC values of 0.786 for Irinotecan and 0.818 for Dasatinib.

## Discussion

There are two key steps for network-based method, i.e., the construction of cell line and drug similarity networks by different types of data and an effective model to execute the prediction. Our method improved the above two steps through an intuitive weighted model which captured different contributions of all available cell line-drug responses. Instead of selecting large plenty of genomic features and making prediction for each drug independently, our model used only two parameters to predict responses for all drugs. This not only decreases the risk of overfitting, but also significantly reduces the computational consumption.

As we all know, a main challenge of computational prediction models is how to achieve good performance with low computational consumption. One may take the following efforts to further improve the performance of the model. First, we can integrate other important information, such as copy numbers, gene mutations, drug resistance and transcriptomic signatures of drug sensitivity into the cell line-drug network to get new knowledge. Second, we could further decrease the computational cost by selecting a few informative genes with respect to drug response to construct cell line similarity network instead of using all genes.

## Conclusion

We built a simple computational model to comprehensively predict anticancer drug responses. One of the main contributions is to provide a technique to predict the response of “new drug to new cell line”*.* Moreover, besides inputting missing values of drug response data, our model could also predict responses of a new drug to existing patients (cell lines), available drugs to a new patient, or even new drugs to new patients. These are more helpful in real clinical practice.

## Additional files

Additional file 1: **Table S1.** The distance matrix of drugs in CCLE dataset. (XLSX 37 kb)

Additional file 2: **Table S2.** The distance matrix of drugs in GDSC dataset. (CSV 11 kb)

Additional file 3: **Table S3.** Prediction accuracy, sensitivity, specificity and goodness of fit for CCLE dataset. (CSV 6 kb)

Additional file 4: **Table S4.** Prediction accuracy, sensitivity, specificity and goodness of fit for GDSC dataset. (XLSX 34 kb)

## Abbreviations

AUC: Area under the ROC curve
CCLE: Cancer cell line encyclopedia
CDCN: Cell line-drug complex network
CSN: Cell line similarity network
DSN: Drug similarity network
GDSC: Genomics of drug sensitivity in cancer
IC50: The concentration of an anticancer drug to kill half cancer cells
NCI: National cancer institute
NRMSE: Normalized root mean square error
RF: Random forest
RMSE: Root mean square error
ROC: Receiver operating characteristic
SVR: Support vector regression
